# Neuromuscular adaptations after a rehabilitation program in patients with chronic low back pain: case series (uncontrolled longitudinal study)

**DOI:** 10.1186/1471-2474-14-277

**Published:** 2013-09-24

**Authors:** Arnaud Dupeyron, Christophe Demattei, Pascal Kouyoumdjian, Olivier Missenard, Jean Paul Micallef, Stéphane Perrey

**Affiliations:** 1 Movement to Health (M2H), Montpellier-1 University, Euromov, 700, Avenue du Pic Saint-Loup, 34090, Montpellier, France; 2 Fédération de Médecine Physique & de Réadaptation, CHU Nîmes, Place du Pr R. Debré, 30029, Nîmes cedex 9, France; 3 BESPIM, CHU Nîmes, Place du Pr R. Debré, 30029, Nîmes cedex 9, France; 4 Service de Chirurgie Orthopédique, CHU Nîmes, Place du Pr R. Debré, 30029, Nîmes cedex 9, France; 5 INSERM ADR 08, 60 rue de Navacelles, 34394, Montpellier Cedex 5, France

**Keywords:** Low back pain, Rehabilitation, Electromyography, Motor skills, Postural perturbation

## Abstract

**Background:**

To investigate the impact of a short-term multimodal rehabilitation program for patients with low back pain (LBP) on trunk muscle reflex responses and feedforward activation induced by postural perturbations.

**Methods:**

Case series (uncontrolled longitudinal study). Thirty chronic patients with LBP (21 women and 19 men, mean age 42.6 ± 8.6 years, mean weight 73 ± 14 kg, mean height 174 ± 10 cm) were included. The intervention consisted in a 5-day program including therapeutic education sessions (360 min), supervised abdominal and back muscle strength exercises (240 min), general aerobic training (150 min), stretching (150 min), postural education (150 min) and aqua therapy (150 min). Feedforward activation level and reflex amplitude determined by surface electromyographic activity triggered by postural perturbations were recorded from abdominal and paraspinal muscles in unexpected and expected conditions. Subjects were tested before, just after and again one month after the rehabilitation program.

**Results:**

No main intervention effect was found on feedforward activation levels and reflex amplitudes underlining the absence of changes in the way patients with LBP reacted across perturbation conditions. However, we observed a shift in the behavioral strategy between conditions, in fact feedforward activation (similar in both conditions before the program) decreased in the unexpected condition after the program, whereas reflex amplitudes became similar in both conditions.

**Conclusions:**

The results suggest that a short-term rehabilitation program modifies trunk behavioral strategies during postural perturbations. These results can be useful to clinicians for explaining to patients how to adapt to daily life activities before and after rehabilitation.

## Background

In spite of extensive research efforts, the etiology of low back pain (LBP) remains unclear. Also studies on prognostic factors for symptom chronicity and treatment effect have reported conflicting results [1]. However, in order to design rehabilitation programs, clinicians must understand how changes in trunk neuromuscular control can be adapted or else be considered as adverse pain effects contributing to chronicity [2].

Neuromuscular impairments associated with chronic low back pain (LBP) have been extensively described [3-6]. It is well known that force and endurance in trunk extensors [7] are altered in patients with chronic LBP. Intervertebral disc or ligamentous lesions can alter the coupling between stabilizing muscles [8] resulting in a delayed [9] or reduced [10] activation of deep back muscles, whereas superficial ones [5] or abdominal muscles [11] exhibit higher activation levels than in healthy subjects. Moreover, feedforward activation of trunk muscles disappears or is delayed in rapid arm movements in patients with chronic LBP [12,13] but exhibits a greater activation before transient force perturbations [14]. It is interesting to note that reflex adaptations may help differentiate patients with LBP from healthy subjects [15]. For example, the flexion-relaxation phenomenon has been reported as lacking in most patients with chronic LBP [16]. However, the various pain models applied in studies on trunk muscle control are still insufficiently predictive of these behavioral changes [4] and the notion that neuromuscular changes can be functional in order to maintain stability and reduce loading on injured tissues, remains an hypothesis.

Multidisciplinary rehabilitation programs and exercises have shown positive effects on pain, trunk functions and ability to return to work [17-19]. Based on the biopsychosocial model of chronic LBP management, clinical evaluations are based on physical impairments, restoration of physical activities and voluntary participation. Although trunk muscle behavior is altered in patients with chronic LBP, neuromuscular parameters have rarely been used as dependent variables, and rehabilitation-induced changes for these parameters are only expected. It has been shown that exercises addressed to chronic LBP patients were able to improve maximal (voluntary) trunk muscles activation [20] or modify automatic responses [21] such as the flexion relaxation phenomenon [22]. Skilled training that aimed to consciously activate one muscle independently from others has been shown to be effective for postural activation of abdominal [23] and back muscles [24]. On the other hand, motor training that aimed to produce global activation (co-contraction) did not restore the control of abdominal muscles [25]. Although it has been suggested that back pain-induced alterations in reflex amplitudes and delays [12] may contribute to the recurrence of LBP, it remains unclear whether rehabilitation programs can modify these reflex patterns.

The aim of this uncontrolled longitudinal study was to investigate the immediate and short-term effects of a short-term multimodal rehabilitation program on trunk postural reflex and anticipatory behaviors. We worked on the hypothesis that a 5-day program could induce changes in the feedforward activation and reflex amplitudes of trunk muscle after a postural perturbation.

## Methods

### Study design

We conducted a monocenter uncontrolled longitudinal study. Clinical and neuromuscular parameters were collected before and after a 5-day multimodal rehabilitation program specifically designed for patients with chronic LBP.

### Participants

All patients seen in the local spine care center were referred by their family physicians for pain lasting for more than 3 months and refractory to conventional treatment. Exclusion criteria were: age < 18 years, spine surgery within the last year before the study, obvious secondary benefits (work-related, insurance) or having previously attended a multimodal rehabilitation program. The study was approved by the regional ethics committee.

### Intervention

This multimodal program lasted 5 days as part of an outpatient program in a specialized spine care center. Each patient was enrolled in a therapeutic group of 4 to 6 patients. The intervention consisted in six 1-hour sessions of patient therapeutic education (Back Book) [26] in individual and group work sessions, asso-ciated with supervised physical rehabilitation training consisting of abdominal and back muscle strength exercises (240 min), general aerobic training (150 min), stretching (150 min), postural and movement education (150 min) and aqua therapy (150 min). Subjects were encouraged to repeat the exercises at home after the multimodal program.

### Assessments

Clinical Assessments: Anthropometrics (weight, height, body mass index), symptoms and duration of sick-leave were collected at day (D) 1. Using a visual analog scale (VAS) we assessed lumbar and leg pain, Schober test and fingertip test were used for trunk flexibility at D1 and again at D5 (end of the multimodal rehabilitation program) and D30. Endurance tests (Shirado-Ito and Sorensen) for abdominal [27] and paraspinal muscles [28] were conducted at D1 and D30. Both tests were carried out according to the authors’ recommendations: prone position for the Sorensen test and supine position with hips and knees flexed at 90° for the Shirado-Ito test. Patients were encouraged to maintain a horizontal prone position during the Sorensen test and flex slightly their trunk during the Shirado-Ito test in order to avoid contact with the table.

Various self-reported questionnaires were presented to the patients. The Roland-Morris score [29] was applied for disability assessment. The Dallas pain questionnaire assessed four domains of daily life affected by low back pain: daily activities, work and leisure activities, anxiety and depression status as well as social interest [30]. The Fear-Avoidance Beliefs Questionnaire (FABQ) evaluated patient’s beliefs in relation to the impact of physical activity and work on their LBP [31]. All questionnaires were completed at D1 and D30 to detect short-term changes.

Trunk reflex assessments were designed as such: subjects held a box (35 cm x 50 cm x 40 cm, 350 g) in front of them in an upright position with their upper arms positioned vertically and forearms positioned horizontally. While the subject was waiting in this upright position, 18 sudden downward perturbations were initiated each 10 seconds. These perturbations consisted in the drop of a foam ball (2 kg) into the box at a constant height of 40 cm. To avoid a possible learning effect, three warning conditions occurred in random order: not expected (NE), anticipated by verbal information before release (E) and self-triggered release (ST). In condition E, the evaluator counted back from 3 before releasing the ball and in condition ST, the patient had to say “top” and the foam ball was immediately released (<1 s). Six repeated measures were taken for each condition. All subjects were blinded to the perturbation by a large opaque curtain placed between the subject and the box. An accelerometer (Freescale, Arizona, USA) mounted on to the box was used to determine the perturbation time. To normalize reflex amplitude response, maximal voluntary activation (EMG max) was assessed for each muscle during a maximal voluntary contraction (MVC) test, before performing the tests. To achieve a 5-second maximal abdominal activation, subjects were asked to pull on a cable mounted onto a harness fitted on the subject’s thorax (T8) and connected to a strain-gauge type dynamometer (nominal load 1000 N, Captels, France) while lying down on a table with knees and hips flexed at 120° and 60°, respectively; feet were attached to the table by straps. For MVC determination in the paraspinal muscles, subjects pulled on the cable in an upright position on the table with their knees straight and hips flexed at 30°. Two attempts were performed in each position. All electromyographic (EMG) assessments (see below) were performed at D1, D5 and D30 to evaluate the sustainability of neuromuscular adaptations, if any.

### Electromyography

EMG activity (Biopac MP100, Systems Inc., Santa Barbara, CA; 16 Bits AD conversion, band pass filtered 10–500 Hz, amplification X 2000, input impedance 10MΩ, CMRR > 90 dB) was recorded at a sample rate of 1000 samples/s, from the left side of the lumbar *erector spinae* (LES), *obliquus externus* (EO) and *rectus abdominis* (RA). Electrodes were placed lateral to L3 spinous process for LES (Longissimus) at 3 cm; lateral to the umbilicus for RA also at 3 cm, and at the crossing point of the horizontal line going through the navel and the vertical line passing through the anterior superior iliac crest for OE. Pairs of bipolar (inter electrode distance: 2.5 cm) self-adhesive Ag/AgCl surface electrodes (Contrôle Graphique Medical, Brie-Comte-Robert, France) were placed after slight skin abrasion and cleaning to reduce skin impedance under 10 kΩ. Cables were fixed on the body of each participant to minimize movement artifact.

### Data analysis

**Figure 1:**
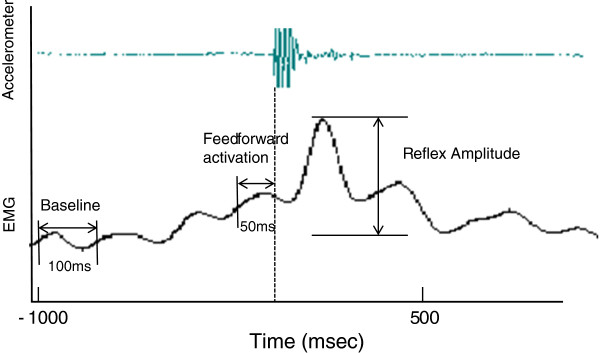
**Sample reflex data illustrating how baseline, feed forward and reflex amplitude were determined.** Baseline EMG activity was measured 1 second before perturbation during 100 ms. Feedforward activation was determined 50 ms before the perturbation. Reflex amplitude was quantified as the peak magnitude of normalized EMG.

EMG signal were recorded and analyzed with Scilab (5.1.1, Paris). After rectification the signal was dual low-pass filtered at 2.5 Hz (first-order Butterworth filter) and normalized with EMG max. The goal of this analysis was to obtain the maximal amplitude of the EMG reflex response. Based on pre-testing and dedicated literature we smoothed the EMG signal in order for it to be as reproducible as possible [32]. EMG max was calculated over a 1-second period at the MVC plateau [33]. Reflex response was recorded at 150 ms after the perturbation [6] and EMG activity baseline was subtracted before normalization. Baseline EMG activity corresponded to the mean activation over 100 ms, one second before the perturbation occurred. The feedforward activation level was determined as t mean EMG activation over the last 50 ms before the onset of the perturbation. Onset of reflex response was automatically calculated using custom algorithms in Scilab and we considered that a muscle response occurred when EMG signal ≥ threshold of 3 standard deviations above baseline (feedforward activation level) [34]. Reflex amplitude was quantified as the peak magnitude of normalized EMG. Accelerometer data were collected at 1000 samples/s and band-pass filtered. Maximum amplitude of acceleration was considered as the beginning of the perturbation. Figure 1 shows a typical try-out with filtered data and an indication of times for measuring feedforward activation, onset of reflex response and its amplitude.

### Statistics

**Figure 2:**
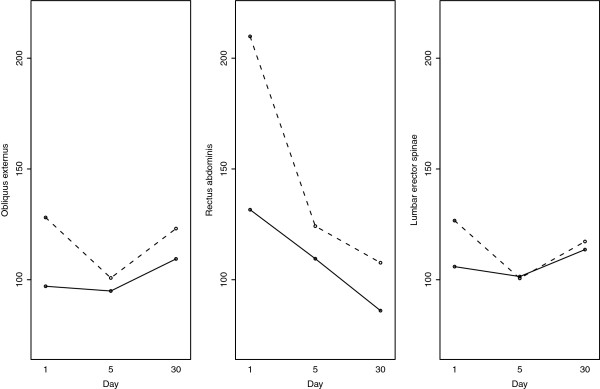
**Normalized reflex amplitude: interaction plot for EMG measures for the three times (D1, D5 and D30) and in the two main conditions (E, NE).** Represented values are expressed as non-transformed means: full line for expected (E) condition and dotted line for non-expected (NE) condition.

**Figure 3:**
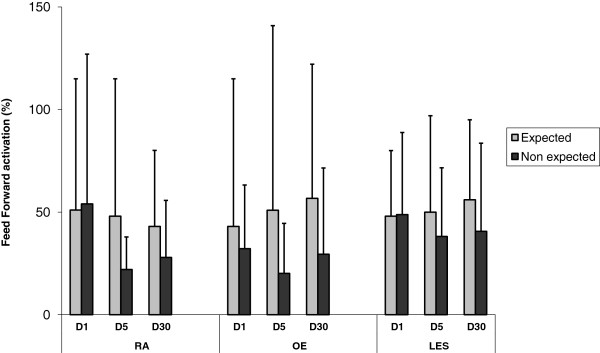
**Feedforward gain measured for each muscle over time.** D: day; RA: rectus abdominis, OE: obliquus externus; LES: lumbar erector spinae. Feedforward gain is calculated as a percentage of variation: % variation = EMG amplitude _t – 0.05 sec_ – EMG amplitude _t-1sec_ / EMG amplitude _t-1sec_ * 100.

Statistical analyses were performed with R software version 2.9.2 (R Development Core Team, Vienna, Austria). For physical and self-reported evaluations, Student t-tests for repeated measures were used. EMG analysis was computed with Box-Cox transformation to normalize data and increase applicability and usefulness of the estimation procedure on structured data throughout a mixed linear model adjusted for each muscle. Therefore, we cannot add units as results come from the mathematical model (Box-Cox transformation) used for this experiment (see Figures 2 and 3). The subject was considered as a random effect in order to take into account repeated intra-individual measures. Warning conditions (E, NE and ST), test date (D1, D5 and D30) and their interactions were considered as explanatory variables of the mixed model. ST and D1 were respectively considered as reference modalities for warning conditions and test date in the models. This interaction was represented on a graph. Wilcoxon matched pair test was used for post-hoc comparisons. Type I error rate was set at 0.05.

## Results

Thirty non-specific patients with chronic LBP (19 men, 11 women) were recruited after having signed an informed consent. All patients but one completed all three evaluations. Patient characteristics are listed in Table 1.

**Table 1:** Population at inclusion (mean ± standard deviation)

	**Global (n = 30)**	**Men (n = 19)**	**Women (n = 11)**
Age (years)	42.6 ± 8.6	42.2 ± 9	43.4 ± 8
Weight (kg)	73 ± 14	80 ± 13	62 ± 7
Height (cm)	174 ± 10	180 ± 6	163 ± 5
Body Mass Index	24 ± 4	25 ± 4	23 ± 3
Duration of pain (months)	10 ± 8	10 ± 9	9 ± 9
Sick leave (months)	5 ± 6	5 ± 6	6 ± 6
Lumbar Pain (VAS)	4.1 ± 2.4	3.9 ± 2.2	4.5 ± 2.7
Leg Pain (VAS)	1.6 ± 2.2	1.2 ± 1.7	2.3 ± 2.7

### Clinical data

Patients showed significant back pain improvements at D5 with clinical significance (-2.1 points on the VAS) at D30. Result of the Schober test remained constant whereas the fingertip-to-floor distance had decreased significantly (D1, 18.3 ± 14 cm; D5, 7.5 ± 10.3 cm; D30, 6.9 ± 8 cm). Abdominal and extensor endurance and MVC were significantly increased at D30 and only the MVC was significantly increased at D5. Results are listed in Table 2.

**Table 2:** Force and endurance parameters (values as mean ± standard deviation)

	**Mean ± standard deviation**		
	**D0 N = 30**	**D5 N = 30**	**D30 N = 29**
Sorensen test (s)	63.9 ± 46.3		101.5 ± 50.7 ‡
Shirado-Ito test (s)	48.7 ± 48.2		82.1 ± 60.7 ‡
MVC Abdominals (Kg)	21.7 ± 11.1	27.8 ± 13.3 *	32 ± 17.5 ‡
MVC Erectors (Kg)	25.4 ± 11	34.6 ± 16.9 *	37.9 ± 17.4 †

*p < 0.05, †p < 0.01, ‡p < 0.001; *MVC*: maximal voluntary contraction.

### Self-reported questionnaires

All disability measures, but two, showed significant improvement. Daily activities demonstrated a 26% (Dallas) to 49% (Eifel) improvement, work and leisure activities 30%, and social interest 3.9%. For anxiety-depression status, the overall improvement was validated by the Dallas score (-40.3%). Regarding patient beliefs, only physical activity fear-avoidance decreased, work effect remained constant.

### Muscle activation

In order to limit the learning effect induced by repeated perturbations, only the three last perturbations for each condition were used in the analysis.

### Reflex amplitude

The intervention did not impact reflex amplitude (no main effect). However, a significant main effect was found for NE condition, where reflex amplitudes were higher than in E condition. When a NE warning condition x epoch interaction was present, post-hoc tests revealed a decreased reflex amplitude in NE condition after the program (D5) for OE (P = .02) but not for LES (P = .06), which disappeared at D30 (Figure 2).

### Feedforward activation

There was no significant main effect of the therapeutic intervention at any of the three epochs. Variation percentage of feedforward activation levels varied between 20% and 32% in NE condition vs. E condition between 20 and 32% vs. 43 and 56% compared to baseline for OE, between 22 and 54% vs. 42 and 51% for RA and between 38 and 48% vs. 48 and 56% for LES muscles. Feedforward activation levels are represented in Figure 3. There was a main difference between NE condition and ST condition for all muscles (OE, P = .002, RA, P < .001, LES, P < .001). In addition, when a warning condition x epoch interaction was present, post-hoc tests revealed a decreased feedforward activation after the program with differences in NE condition between D1 and D5 for OE (P = .02) but not for LES (P = .09) and between D1 and D30 for RA (P = .04). Graphically, we noted a tendency to decreased feedforward activation in NE condition over time whereas in E condition, abdominal feedforward activation was constant and erectors feedforward activation was slightly increased (Figure 3).

## Discussion

This uncontrolled longitudinal study y was designed to observe clinical and neuromuscular adaptations in patients with chronic LBP after a multimodal rehabilitation program. Physical parameters and self-reported disabilities had significantly improved while no significant changes across each condition and over time were detected. In addition, our results suggest that the neuromuscular responses to postural perturbations in abdominal muscles are differentially altered by this type of program according to warning conditions (expected vs. non-expected perturbation) with a supposed shift in motor control strategy. These preliminary results suggest a potential reversibility of neuromuscular adaptation induced by chronic LBP [2,35] and probable mechanism underlying functional restoration in accordance with the fear-avoidance models [36].

### Changes in physical performance

Intervention studies in chronic LBP generally do not unveil different clinical outcomes between specific exercises (stabilization, or skilled-cognitive activation) and general training (strength training, unloaded training) [37,38]. The present study highlighted improvements for all physical parameters with increased strength ranging from 22% to 32% for abdominal muscles and 27% to 33% for back muscles at D5 and D30 respectively, increased endurance (+41% and 37% for abdominal and back muscles respectively at D30) as well as increased flexibility. Dynamic rehabilitation programs with similar duration and physical intensity were reported as providing approximately the same improvements [39]. Since no specific exercises such as strength training, endurance or stretching were imposed during our 5-day multimodal program, improvements observed in patients with chronic LBP patients cannot be due to a specific training effect, but rather are more likely related to motor skill recovery following training and/or by reduced fear of pain during movement [40]. Although the correlation between pain-related fear and physical performance has been already demonstrated [41] we did not unveil such a relationship between endurance or strength and FABQ test. Nevertheless, it is not possible to generalize avoidance of physical activities (as reported in self-questionnaires) daily live situations. Moreover, it is possible that better performances were due to decreased pain rather than fear-avoidance. However no correlation was found between performance and pain intensity when compared to other studies [42]. The mechanisms underlying such recovery processes cannot be detected by the usual tests (FABQ is not considered as a good tool for outcome measures [3]) and further explorations are needed in the behavioral and sensorimotor fields.

### Changes in anticipation behavior (feedforward activation)

Anticipatory adjustments increase the load on the spine as muscles offset the imbalance and thereby limit paraspinal reflex occurrences. Lavender et al. showed on a small sample of healthy subjects the potential role of experience (repetitive sudden load paradigm) in anticipatory strategies [42]. Feedforward adjustments and paraspinal muscles recruitment are altered in patients with chronic LBP compared to healthy controls [43]. To our knowledge, this study is the first to investigate changes over time in feedforward trunk muscle responses and in different conditions for a population of patients with chronic LBP. Although one can expect trunk strengthening to improve endurance or strength abilities, it seems less plausible for such training to improve paraspinal feedforward control in just over a few days in patients with chronic LBP. Instead, the elements described above advocating non-specific training effects are probably valid for motor skills such as postural adjustments [44]. It has been recently demonstrated that isolated training of *transversus abdominis* muscle alters anticipated postural adjustments over short-term [23,24] and longer term [44]. Nevertheless, the results of our study, in accordance with others [21,25] showed no main training effects on trunk muscle recruitment after training programs. However, in the present study, the difference between both conditions (expected and unexpected) became significant after the intervention. Before the rehabilitation program, patients with chronic LBP exhibited a uniform neuromuscular behavior with similar feedforward activation across conditions, as though the subjects were not influenced by the warning information. After the 5-day multimodal program, feedforward activations were different between the warning conditions (see Figure 3), and patients seemed to reduce feedforward control in the unexpected condition vs. expected condition and this for most recorded muscles (see Figure 3). In non-expected conditions, Stokes et al. [14] demonstrated a larger feedforward activation in patients with LBP compared to healthy subjects. Some authors have defined motor control strategies in patients with chronic LBP as shifting from feedforward to feedback control [45]. Our results can be interpreted as new feedforward strategy post rehabilitation. Indeed, feedforward control might better promote spinal stability when appropriately timed (E condition).

### Amplitude response changes

Sudden loading is considered a risk factor for low back pain [46]. Unexpected loading incidents are difficult to predict but in healthy subjects it was reported that training can modify the response patterns to adjust to sudden loading [47,48]. In chronic LBP patients with chronic LBP, back muscles activity is increased during trunk movements [14], or during imbalance [11], suggesting a compensation mechanism to restore and maintain balance [48]. Moreover, lumbar EMG responses are increased when subjects are unaware of perturbations [49]. It is interesting to note that, in this population, these responses decrease when the timing of the perturbation is known [50]. Our data unveiled the same differences in between conditions before the rehabilitation program. However, after the program, trunk muscle responses switched from overreacting in NE (compared to E condition) to a similar response pattern after the program, regardless of conditions (Figure 2). These results, in part, match findings from previous studies. Magnusson et al. demonstrated in a small sample of patients with chronic LBP that a 2-week rehabilitation program caused a decreased amplitude response for spinal muscles [51]. Despite no significant training effect, Pedersen et al. also suggested improvements for workers after a 9-week training in non-expected trunk loading conditions [47]. It is also possible that this type of rehabilitation program improves co-activation allowing for a lesser activation. This suggests a common control of all antagonist muscles, which end up working together [52]. Therefore, one can expect that responses to sudden load occurring in daily life activities, yield less pain than random trivial movements. Finally, in order to design specific rehabilitation programs for this population, it is essential to address the relationship between motor representation and dynamic stability.

### Study limits

The major limit of the present study was the absence of a control group or control session to validate the rehabilitation related effect and neuromuscular changes. However, this uncontrolled longitudinal study was only designed to explore behavioral changes and presence of neuromuscular adaptations, if any, after a short-term rehabilitation program. Patients could have modified their postural strategy as a learning effect. The random process proposed in this study associated with the exclusion of first trials in each testing condition may have lowered such bias. Finally, the normalization process limited the amplitude gain since activation during MVC was greatly increased but repeated measures needed to be compared as accurately as possible. Moreover, since the goal of this study was to assess muscle recruitment for a given task before and after the rehabilitation program, MVC normalization was probably the best way to reduce inter-subject variability [53].

## Conclusion

This non-specific rehabilitation program aimed patients with chronic LBP quickly improved trunk performances. This study suggests that rehabilitation programs may change the way patients with chronic LBP adapt to postural perturbations according to various warning conditions. After the rehabilitation program, feedforward paraspinal responses became greater between conditions whereas evoked responses remained similar, thus bringing up a shift in behavioral strategies. These results need to be confirmed in a randomized controlled trial.

## Abbreviations

CNS: Central nervous system; CLBP: Chronic low back pain; EMG: Electromyography; EO: External oblique; LBP: Low back pain; LES: Lumbar erector spinae; MVC: Maximum voluntary contraction; RA: Rectus abdominis; E: Expected; N: Unexpected; SEMR: Short educational and multidisciplinary rehabilitation.

## Competing interest

The authors declare that they have no competing interests.

## Authors’ contributions

AD, JPM and SP conceived the study; AD and PK performed the measurements; OM carried out the electromyographic analysis; CD performed the statistical analysis; all authors read and approved the manuscript.

## Pre-publication history

The pre-publication history for this paper can be accessed here:

http://www.biomedcentral.com/1471-2474/14/277/prepub
